# Transthoracic echocardiography-guided subaortic ventricular septal defect closure in infants: a case report

**DOI:** 10.3389/fcvm.2025.1647073

**Published:** 2025-09-24

**Authors:** Radityo Prakoso, Rina Ariani, Yovi Kurniawati, Brian Mendel, Oktavia Lilyasari

**Affiliations:** ^1^ Division of Pediatric Cardiology and Congenital Heart Disease, Department of Cardiology and Vascular Medicine, National Cardiovascular Centre of Harapan Kita, Universitas Indonesia, Jakarta, Indonesia; ^2^ Division of Non-invasive Diagnostic and Cardiovascular Imaging, Department of Cardiology and Vascular Medicine, National Cardiovascular Centre of Harapan Kita, Universitas Indonesia, Jakarta, Indonesia; ^3^ Department of Cardiology and Vascular Medicine, National Cardiovascular Centre of Harapan Kita, Universitas Indonesia, Jakarta, Indonesia

**Keywords:** case report, subaortic VSD, small body weight, TTE, zero-fluoroscopy

## Abstract

**Introduction:**

Subaortic VSD, while similar to perimembranous defects, pose a higher risk for aortic valve insufficiency and AV block. This case aims to assess the safety and efficacy of percutaneous subaortic VSD closure in infants under 10 kg using transthoracic echocardiography-only guidance.

**Case presentation:**

A one-year-old infant, 8.9 kg, was scheduled for subaortic VSD closure due to concerns of failure to thrive. Percutaneous closure was performed using a retrograde transarterial approach with a 7/5 mm Konar-MF VSD Occluder (Lifetech) under TTE guidance. Apical 5-chamber view showed smallest VSD diameter 3.8 mm. 3.5/5F Guiding JR catheter with soft hydrophilic wire were then maneuvered to descending aorta in subxiphoid 12 o'clock view, suprasternal short axis view and positioned just above the aortic valve. Catheter was then entered to the LV shown by parasternal long axis view. 3.5/5F Guiding JR catheter was then crossed the subaortic VSD in parasternal short axis view. The Konar-MF VSD Occluder (Lifetech) No. 7/5 mm was deployed assisted by apical 5-chamber view. Device detachment was then evaluated in parasternal short axis view showing no residual shunt. At six-month follow-up, the device was well seated, and the symptoms subsided.

**Conclusions:**

Our case underscores that zero-fluoroscopy TTE-only percutaneous subaortic VSD closure is feasible in selected patients under 10 kg with no major complications.

## 1 Introduction

Subaortic ventricular septal defects (VSDs) resemble perimembranous VSDs anatomically, but physiologically, they have a higher tendency for right coronary cusp prolapse, which can lead to aortic valve insufficiency (1). Closing a subaortic VSD poses unique challenges due to its proximity to the aortic valve (2). Surgical closure remains the standard treatment for subaortic VSD closure, but it is associated with notable morbidity, as patients often require intensive care unit (ICU) admission and blood transfusions (1, 2). Additionally, smaller infants face an increased risk of atrioventricular (AV) block (3). A meta-analysis involving over 6,300 patients undergoing transcatheter VSD closure reported a rate of complete AV block (cAVB) comparable to surgical outcomes, at 1.1% (4).

Percutaneous closure of subaortic ventricular septal defects (VSDs) in infants weighing less than 10 kg is challenging (3), with a higher risk of procedural failure, device-related complications, and adverse events, often requiring extended fluoroscopy times (5). Small infants are particularly susceptible to the harmful stochastic and deterministic effects of radiation. To address these risks, various strategies have been developed to reduce radiation and contrast exposure, including zero-fluoroscopy or radiation-free techniques (6, 7). Despite these advancements, the procedure remains technically demanding in low-weight infants due to their smaller femoral vessels, with arteriovenous loop formation potentially causing rhythm disturbances and hemodynamic instability (3). This study aims to assess the efficacy and safety of percutaneous subaortic VSD closure in infants under 10 kg, utilizing transthoracic echocardiography (TTE)-only guidance.

## 2 Case presentation

A one-year-old female infant, weighing 8.9 kg, was scheduled for ventricular septal defect (VSD) closure. She had no history of feeding difficulties, recurrent respiratory tract infections, or febrile episodes. However, her parents reported concerns about failure to thrive. A heart murmur was first detected during a physical examination at three days of age. The patient was born at 38 weeks of gestation via cesarean section due to maternal pre-eclampsia, with a birth weight of 3,790 g. There was no family history of congenital heart disease. On examination, the patient was alert with a heart rate of 112 beats per minute, a respiratory rate of 26 breaths per minute, a temperature of 36.8°C, and oxygen saturation of 98%. Cardiac auscultation revealed normal S1 and S2 heart sounds, accompanied by a grade III/VI pansystolic murmur at the left lower sternal border. No gallop rhythms were detected. She had been previously prescribed captopril 6.25 mg three times daily.

**Figure 1:**
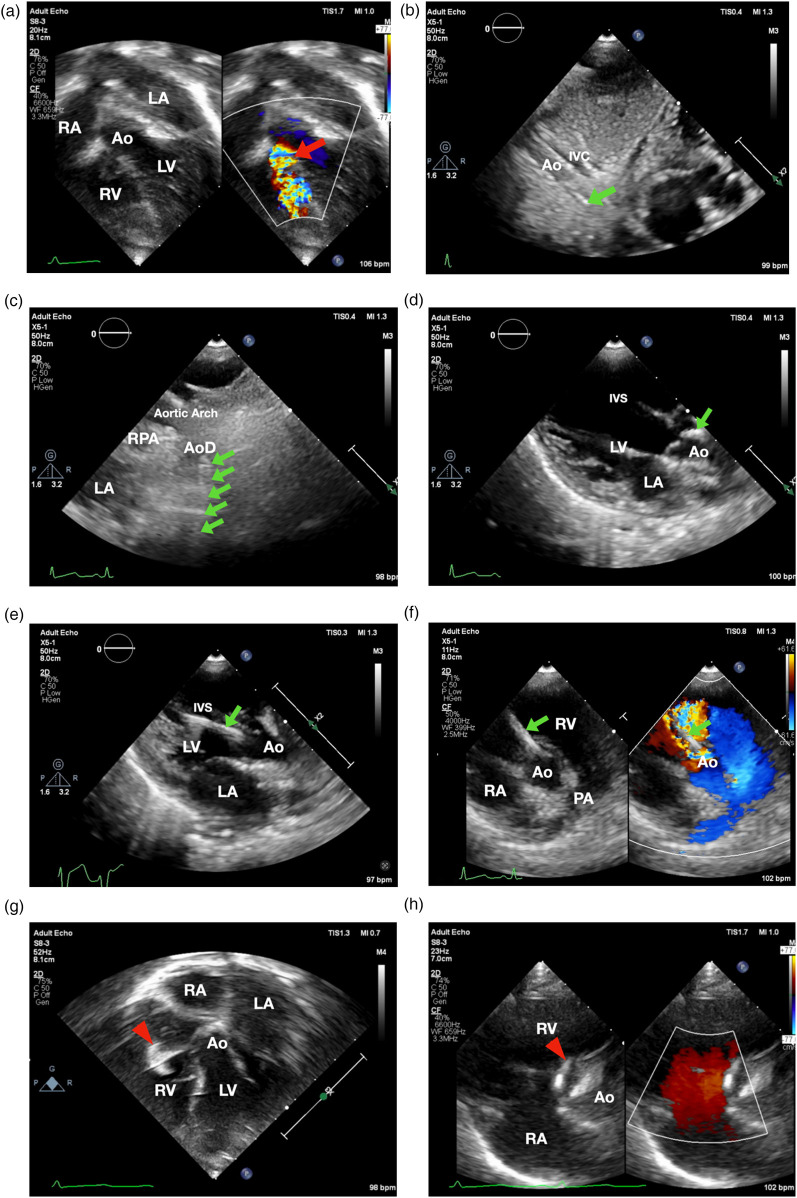
Transthoracic echocardiographynnn-guided only subaortic VSD closure in infants. **(a)** Apical 5-chamber view showed smallest VSD diameter 3.8 mm (showed by the red arrow). **(b)** 3.5/5F Guiding JR catheter with soft hydrophilic wire were entering descending aorta in subxiphoid 12 o'clock view (indicated by the green arrow) **(c)** suprasternal short axis view. **(d)** Guiding catheter was then positioned just above the aortic valve. **(e)** Catheter entered LV as shown in parasternal long axis view. **(f)** 3.5/5F Guiding JR catheter crossed the VSD in parasternal short axis view. **(g)** The Konar-MF VSD Occluder (Lifetech) No.7/5 mm (indicated by red arrowhead) was deployed in apical 5-chamber view. **(h)** Device detachment was evaluated in parasternal short axis view showing no residual shunt. Ao, aorta; AoD, descending aorta; IVC, inferior vena cava; IVS, interventricular septum; LA, left atrium; LV, left ventricle; PA, pulmonary artery; RPA, right pulmonary artery; RA, right atrium; RV, right ventricle.

The patient underwent the procedure under sedation. Preprocedural apical 5-chamber view revealed a subaortic ventricular septal defect (VSD) with a membranous septal aneurysm (MSA), left-to-right shunt, with VSD exit diameter of 3.8 mm, and an inlet diameter of 6–7 mm (Figure 1a). There was an adequate distance between the VSD and the tricuspid and aortic valves. Mild mitral regurgitation was noted without evidence of aortic regurgitation. Based on these findings, percutaneous VSD closure was planned using a retrograde transarterial approach with TTE-only guidance.

Aseptic and antisepsis were prepared in the left and right femoral regions. The right femoral artery was punctured, and a 4/5F slender sheath was inserted. Heparin (800 IU) was administered intra-arterially. A 3.5/5F JR guiding catheter was advanced via the descending aorta, aortic arch, and ascending aorta into the left ventricle using the subxiphoid 12 o'clock view (Figure 1b), suprasternal short axis view (Figure 1c), parasternal long axis view in which the catheter was positioned just above the aortic valve (Figure 1d) and pressure monitoring, with the assistance of a 0.035” Terumo soft hydrophilic wire. The guiding catheter was used to cross the VSD from LV to RV using parasternal long axis view (Figure 1e) and parasternal short axis view (Figure 1f). Hemodynamic measurements showed left ventricular pressure of 80/16 mmHg, right ventricular pressure of 20/6 mmHg, and descending aortic pressure of 71/37 mmHg (mean 49 mmHg). Left ventricular oxygen saturation was 98%. Prophylactic intravenous cefuroxime (400 mg) was administered.

A 5F delivery cable and a Konar-MF VSD Occluder (Lifetech) size 7/5 mm were introduced through the guiding catheter and positioned within the defect. The low-pressure disc was deployed in the right ventricle as shown in inverted apical 5 chamber view (Figure 1g**)** followed by deployment of the high-pressure disc in the left ventricle as shown in parasternal short axis view (Figure 1h).

The total procedural time was 46 min. Postprocedural descending aortic pressure was 73/38 mmHg (mean 51 mmHg) with peripheral oxygen saturation of 100%. No complications were observed. Post procedure, the patient was prescribed captopril 6.25 mg three times daily, aspirin 45 mg once daily, and bisoprolol fumarate 0.125 mg once daily, and cefuroxime 500 mg once daily. At six-month follow-up, TTE showed a well-seated device without residual shunt, and good left and right ventricular function (LVEF 68% and TAPSE 1.5 cm).

## 3 Discussion

### 3.1 Feasibility of subaortic ventricular septal defect closure in infants

Subaortic VSDs are typically perimembranous or involve both semilunar valves, positioned near the conduction system. They are usually restrictive and often associated with right coronary cusp prolapse, leading to aortic insufficiency (1, 2). These defects, commonly crescent-shaped and malaligned, creating turbulence that promotes fibrous membrane formation. Hu et al. (2) defined subaortic VSDs as those with a tissue rim of less than 2 mm below the aortic valve.

According to the 2022 ACC guidelines (8), transcatheter VSD closure is reasonable (Class IIa, Level B) for patients >5 kg with hemodynamically significant defects and suitable anatomy. Our patient had failure to thrive, prompting intervention. However, smaller infants are at greater risk of AV block (3). Early-generation devices were associated with unacceptably high rates of complete heart block, but advancements in device design, particularly softer materials with reduced radial force, have mitigated this concern. We used the KONAR-MF VSD Occluder (Lifetech) which has a softer profile, which result in no total AV block during or after the procedure (8, 9). However, it has high incidence of residual shunt, which typically resolves over time (8–10).

Early reports highlighted challenges in infants <10 kg. Alshahrani et al. (3) reported an 88% success rate in sixteen patients with pmVSD (median weight: 8 kg, defect size: 6 mm). The main challenge is assessing device interaction with the tricuspid and aortic valves before release, especially in subaortic VSD with a deficient aortic rim, which may cause significant aortic regurgitation (AR). AR can result from leaflet impingement, annular distortion, or subaortic membrane configuration with larger devices. Distinguishing leaflet impingement from cable-induced distortion in the retrograde approach requires careful echocardiographic assessment (2, 3, 9, 11). While concerns exist about large sheaths in small femoral arteries, our patient had no vascular compromise.

### 3.2 Zero fluoroscopy transthoracic echocardiography-only ventricular septal defect closure

Percutaneous VSD closure in children <10 kg is often linked to procedural failure, device-related complications, and longer fluoroscopy times (3). Considering this patient was an infant, our team decided to perform VSD closure with no radiation guidance. Conventional TEE probes frequently necessitate the use of general anesthesia because of patient discomfort. The advent of miniaturized TEE probes offers a promising alternative, facilitating routine diagnostic assessments and interventional procedures under minimal sedation (12). In a direct comparison, Papadopoulos et al. (12) demonstrated that the 4D mini-TEE probe provided image quality comparable to that of the standard 6VT-D probe, underscoring its potential utility in pediatric practice. Unfortunately, this probe is not available at our institution; if it were, it could serve as a valuable adjunct for more precise guidance during device closure procedures. Therefore, we performed the procedure using a zero-fluoroscopy technique guided solely by transthoracic echocardiography.

The size of the subaortic VSD was first assessed using the apical five-chamber view. Subsequently, the catheter was advanced from the descending aorta to the aortic arch under guidance from the subxiphoid 12 o'clock view, the suprasternal short-axis view, and the parasternal long-axis view. The catheter was then positioned just before the aortic valve. Once its location was confirmed, the catheter was advanced into the left ventricle, and with careful backward maneuvering, it was successfully directed across the subaortic VSD into the right ventricle, as demonstrated in the parasternal short-axis view. The main challenge in crossing subaortic VSD is when its angle to the interventricular septum is ≤90 degrees.

TTE provides real-time visualization of valve function and hemodynamics, ensuring precise occluder placement and detecting residual shunts. It also minimizes the risk of tricuspid regurgitation by providing real-time guidance for disc positioning, ensuring proper defect coverage while avoiding tricuspid valve and chordae injury (11, 13). In this case, retrograde transarterial approach led to mild post-procedure AR. Compared to the antegrade approach, the retrograde method is simpler and may significantly shorten procedure time. Serial echocardiographic assessments are crucial for identifying the exact AR mechanism, with comparative evaluations before and after device deployment helping distinguish cable-induced from device-related regurgitation (2, 3, 9).

## 4 Conclusions

Our case demonstrates that percutaneous subaortic VSD closure can be successfully performed under TTE guidance in infants without significant complications. Larger, controlled studies are needed to assess the feasibility and long-term outcomes of this approach.

## Data Availability

The original contributions presented in the study are included in the article/Supplementary Material, further inquiries can be directed to the corresponding author.
